# Care and Neurorehabilitation in the Disorder of Consciousness: A Model in Progress

**DOI:** 10.1155/2015/463829

**Published:** 2015-03-29

**Authors:** Giuliano Dolce, Francesco Arcuri, Simone Carozzo, Maria Daniela Cortese, Pierpaolo Greco, Lucia Francesca Lucca, Loris Pignolo, Maria Elena Pugliese, Francesco Riganello

**Affiliations:** Institute S. Anna-Research in Advanced Neurorehabilitation (RAN), 88900 Crotone, Italy

## Abstract

The operational model and strategies developed at the Institute S. Anna-RAN to be applied in the care and neurorehabilitation of subjects with disorders of consciousness (DOC) are described. The institute units are sequentially organized to guarantee appropriate care and provide rehabilitation programs adapted to the patients' clinical condition and individual's needs at each phase of evolution during treatment in a fast turnover rate. Patients eligible of home care are monitored remotely. Transferring advanced technology to a stage of regular operation is the main mission. Responsiveness and the time windows characterized by better residual responsiveness are identified and the spontaneous/induced changes in the autonomic system functional state and biological parameters are monitored both in dedicated sessions and by means of an *ambient intelligence* platform acquiring large databases from traditional and innovative sensors and interfaced with *knowledge management and knowledge discovery* systems. Diagnosis of *vegetative state/unresponsive wakefulness syndrome* or *minimal conscious state* and early prognosis are in accordance with the current criteria. Over one thousand patients with DOC have been admitted and treated in the years 1998–2013. The model application has progressively shortened the time of hospitalization and reduced costs at unchanged quality of services.

## 1. Introduction

Survival and the outcome of subjects in* vegetative state/unresponsive wakefulness syndrome* (VS/UWS) or* minimally conscious state* (MCS) have improved significantly over the last two decades due to the logistic and medical/paramedical resources that have been increasingly made available in dedicated private or healthcare units of developed countries. If given proper care, hydration, and nutrition, subjects in VS/UWS can either evolve into a MCS [1–5] or remain in VS/UWS for months or years; late recovery is not exceptional. About 80% of posttraumatic subjects recover consciousness and over 60% of them are discharged with minor residual disabilities [6–12]. Adequate medical care and rehabilitation plans are mandatory to cope with a complex brain dysfunction such as VS/UWS or MCS and to help promote recovery. Unambiguous diagnosis, early prognosis, and adequate monitoring during treatment are needed in order to optimize treatment, reduce hospitalization, and improve outcome. The Institute S. Anna-RAN has been designed and upgraded since 1998 to fulfill the requirements of subjects with disorder of consciousness (DOC) by transferring advanced technology to a stage of regular operation. The working model and the strategies developed over the years have proved cost-compatible and efficient, with reduced length of hospitalization and duration of the rehabilitation protocols. The model rationale and application are described.

## 2. Patients and Clinical Diagnosis

One of the few Italian structures to treat DOC following acquired severe brain injury, the Institute S. Anna-RAN was first designed to serve a local population of 1,9 million and a large portion of the country DOC subjects. Referrals from other regions have decreased over the years after numerous dedicated healthcare or private units have been made available compliant to the indications by the 2000, 2005, and 2010 national consensus conferences [13–15] and government steering committees on DOC. As a consequence, referrals from local hospitals have increased from 1998 to 2013 from 40% to 90% and the institute patients' population has become regionally representative. Posttraumatic subjects have decreased steadily from 59% to 30% while those in VS/UWS or MCS due to vascular brain injury have increased from 33% to 55%; the average age at admission has increased in parallel from 39 to 58 yrs. Patients are mostly referred from local ICU or neurosurgery units. It should be noted that 24% of them were not in VS/UWS at admission in the 1998–2009 period, possibly because of spontaneous early recovery while awaiting admission; this percentage has increased over the years and prompter or improved assistance after brain injury is conceivable. Admission criteria to the Institute S. Anna-RAN are autonomous breathing, stability of vital parameters, and absence of indication for further (neuro)surgery. Patients are classified as being in VS/UWS or MCS according to the current clinical criteria and the guidelines by the Aspen group [16]. Some of the available evaluation scales are used for a better characterization. In VS/UWS, scores are lower than 25 at the Loewenstein Scale (only used from 1998 to 2008) [17]; between 1 and 2 at the Level of Cognitive Function scale (LCF) [18]; higher than 21 at the Disability Rating Scale. Evolution into a MCS is upon observation of reproducible or sustained behavioral patterns associated with awareness of self or environment and with support by means of the Coma Recovery Scale-Revised (CRS-r) [2, 19]. The CRS-r was not in use until 2002 and was introduced in Italy only later. Subjects admitted to the Institute S. Anna-RAN in 1998–2002 were initially diagnosed as being in VS/UWS with “atypical” VS/UWS or without any consistent behavioral responsiveness; in this regard, the guidelines by the Aspen Neurobehavioral Conference Workgroup were informally followed [20]. The clinical records have been revised for the present study and the diagnosis of VS/UWS and MCS was reformulated according to these guidelines. The introduction of the CRS-r has improved the diagnostic differentiation between VS/UWS and MCS, with higher percentage of MCS subjects correctly diagnosed and better overall classification accuracy than the current clinical criteria alone [21]. Outcome is conventionally assessed by the Glasgow Outcome Scale [22] despite its occasional ambiguities regarding the evaluation of consciousness in VS/UWS and MCS [23]. One thousand and one hundred patients with DOC have been admitted, treated, and discharged in the years 1998–2013 (Table 1).

## 3. Overall Structure and Organization Model

**Figure 1:**
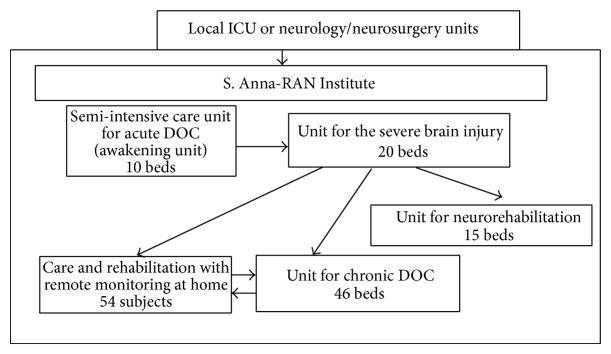
The S. Anna model for the care and neurorehabilitation of brain-injured subjects with DOC.

The institute general architecture has been planned and its units have been sequentially organized in order to guarantee appropriate care and provide advanced neurorehabilitation plans consistent with the clinical condition and meeting the patients' individual needs at each phase of evolution, either spontaneously or following rehabilitation (Figure 1). Treatment is intensive, with the exception of long-term rehabilitation of chronic DOC, and includes sensory stimulation programs based on tactile, olfactory, gustatory, auditory, and visual stimulus conditions when deemed appropriate [24–26].

- Acute VS/UWS subjects are admitted to a dedicated 10-bed semi-intensive care unit (jargonized as the* awakening unit*), with constant temperature and humidity and circulating sterile air. All beds can be moved to intermediate and upright positions to promote the subjects' adaptation to standing and help recover the autonomic balance. After clinical and neuroradiological (TC or MRI) assessment of the brain damage, medical and nursing care is focused on stabilizing the patient's conditions; close monitoring over time helps prevent possible complications (e.g., paroxysmal sympathetic hyperactivity or hydrocephalus). Patients are monitored by conventional procedures as well as by an* ambient intelligence* platform combining traditional and innovative sensors and interfaced with advanced tools for knowledge management and knowledge discovery [27, 28]. The staff working shifts and turnover guarantee an overall 8 hr/day/patient medical and nursing assistance and rehabilitation treatments.
- Subjects emerging from VS/UWS into MCS are moved to a dedicated 20-bed unit, where monitoring is limited to the vital parameters and assistance is provided for a total 7 hr/day/patient. Procedures are activated on each subject in order to favor recuperation (when realistic) or to help compensate for the cortical/subcortical, sensory, or motor disabilities resulting from brain damage.
- A 46-bed unit is dedicated to the long-term care of patients not evolving from a VS/UWS or MCS and unsuitable for discharge or homecare. Patients are monitored over time for stability and possible late recovery, with enforcement of the procedures to prevent disorders or dysfunctions due to chronic hospitalization.
- A model for care and rehabilitation of chronic VS/UWS and MCS subjects at home and with remote professional control has been designed in collaboration with the local healthcare authorities and is operative. Main goals are to facilitate the patients' discharge from hospital and return home as early as possible; to disseminate the Institute S. Anna-RAN expertise and procedures among the collaborating family doctors and recruited nurses; to increase the number of patients under proper treatment to full coverage of the regional needs without increasing the number of dedicated beds/units in public or private structures (in this case, 54 beds of the chronic DOC unit have been successfully transferred to home care); to improve the management of DOC subjects by the regional healthcare organization. To this end, the reliability of the subject's family is tested through a two-week training program and the home environment is carefully controlled and provided with proper healthcare equipment and domotics/informatics support; dedicated physician, nurse, and therapist are recruited and properly trained and their support to the patient and his/her family is scheduled according to the individual's needs.

**Figure 2:**
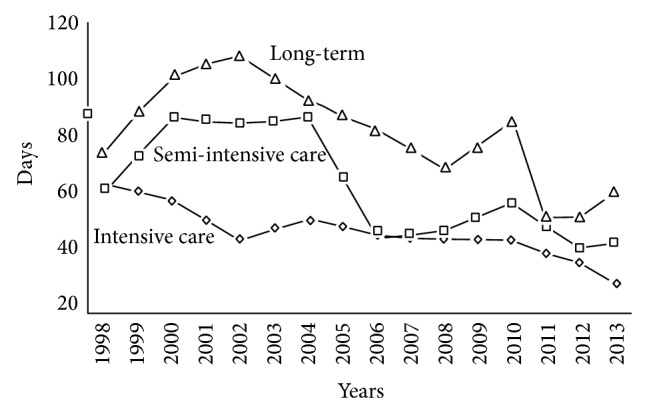
Average time (days) spent in the ICU or neurology/neurosurgery units of local hospitals before admission and in the Institute S. Anna-RAN dedicated units after referral.

The fast turnover between units favors prompt admission to the* awakening unit* and has progressively reduced the length of hospitalization in the ICU or neurosurgery units of local hospitals, with reduced public healthcare commitment and costs for these clinical conditions. It has also promoted the subjects' transfer across the Institute S. Anna-RAN units to meet the individual clinical changes, with optimization of the facilities consistent with each subject's need. The turnover rate has significantly improved with the increasing number of available beds in the long-term unit in 2004-2005 (Figure 2) and the beginning of the home care project. Medical care and rehabilitation at the Institute S. Anna-RAN are supported by the healthcare national service on the basis of the total estimated number of beds available for hospitalization of DOC subjects. The faster turnover between the institute units and the increased discharge rate made possible by home care have significantly increased the number of subjects admitted and treated at unmodified budget and therefore have reduced the costs per subject without detrimental effects on the quality of services.

## 4. Responsiveness in DOC

Neuroimaging has shown that subjects otherwise diagnosed as being in VS/UWS may retain high-level aspects of brain activity across sensory modalities, language and learning dynamics, emotions, or pain. These responses vary in complexity, from activation of primary sensory cortices, to the involvement of associative areas, to activation of cortical-subcortical networks to either mental imagery or distinction of ambiguous/nonambiguous words or figures [29, 30]. However, brain activation reflecting some awareness and cognition has been unambiguously observed in only a small portion of VS/UWS subjects [29] or the residual functions have been regarded as reflecting intact but functionally disconnected cortical modules not necessarily giving rise to phenomenological consciousness [29, 31–33]. Functional assessment by neuroimaging is mostly limited to research, and the diagnosis of VS/UWS and MCS and prognosis remain the responsibility of the clinician, to whom the breakthrough evidence from neuroimaging research adds professional burdens by introducing novel criteria of evaluation of responsiveness not yet fully integrated in the current nosographic criteria for DOC [31, 32].

## 5. Responsiveness and Individual Variability

**Figure 3:**
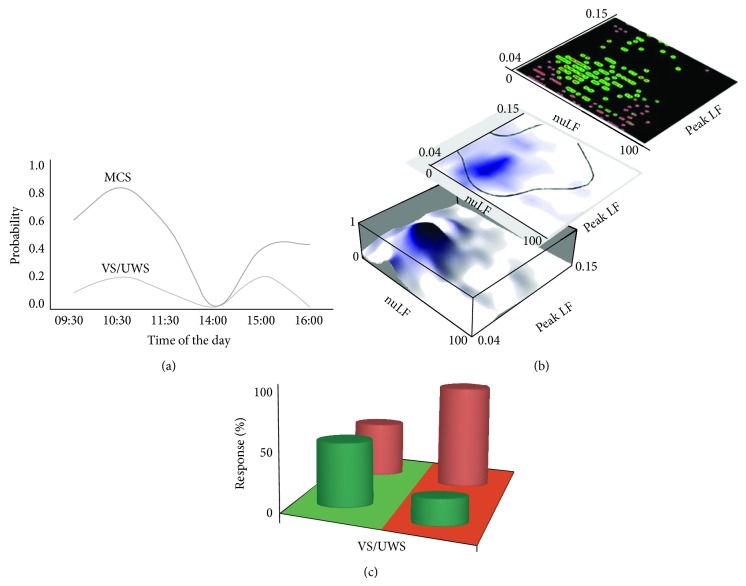
(a) Probability of observing a positive visual pursuit response over time during the day in VS/UWS and MCS subjects repeatedly tested (6 tests/subject/day). Maxima are at approximately 10.00 a.m. and 3.00 p.m., while no response was observed at postprandial time (after Candelieri et al., 2011 [41], modified). (b) Top right: scatterplot of positive visual pursuit responses (green) and no observed responses (red) in a group of DOC subjects versus the values of the HRV descriptors* nuLF* and* pkLF*; middle: support vector machine model predicting the target data values (presence or absence of a pursuit response) to which specific attributes (the HRV descriptors) could be related; bottom left: probability of observing a pursuit response estimated as the relative frequency of response for each subject versus each HRV descriptor (after Riganello et al., 2013 [39], modified). The overall incidence of positive responses or no response at all is summarized in the inset at (c), where the model areas at which a response could or could not be predicted by HRV estimates are in green and red, respectively, and the actual percentage of responses/no responses is reported.

The visual pursuit response is a major CRS-r item observed in 70–80% of subjects in MCS [34–36] and a key marker of evolution from VS/UWS [37, 38]; it has been reported with lower incidence (~20–30%) also in subjects otherwise unambiguously diagnosed as VS/UWS [10, 29, 39, 40]. The controversy on whether this or other responses may indicate residual automatic subcortical activities compatible with, but atypical for, VS/UWS or may rather signal higher order cortical activation and partially recovered consciousness remains unsolved [20]. The incidence of established clinical indicators of responsiveness has proven variable also in the single subject. Multiple testing (6 tests/subject/day) has shown that the incidence of the pursuit response in VS/UWS or MCS is not at random during the day; positive responses were observed more often in the morning than in the afternoon in both VS/MCS and MCS subjects and the probability of observing a response during the day was described by comparable curves with maxima at 10.30 a.m. and 3.00 p.m. and minima at 2.00 p.m. The overall chances of observing a response at least once per day were ~33% and ~62% in the VS/UWS and MCS, respectively [41] (Figure 3). The CRS-r global, visual, and auditory scores were also higher in the morning than in the afternoon in VS/UWS and MCS subjects repeatedly tested during the day and over time [41]. These figures (and the risk of erroneous classification in case of single random CRS-r testing) are in agreement with an estimated misdiagnosis rate between these conditions [21, 31, 42, 43] and thus make a binary distinction unreliable and reduce diagnostic accuracy. The clinical criteria in use to characterize subjects with DOC and predict outcome are to be reconsidered by including individual variability as a possible major independent variable deserving proper consideration.

## 6. Responsiveness and Heart Rate Variability

The measures of heart rate variability (the heart rate fluctuations around the mean value over the time sample, HRV) are regarded as reliable descriptors of the sympathetic/parasympathetic functional interplay and are thought to also reflect brain function [44, 45]. Interest in the bidirectional interaction between the CNS and the autonomic nervous system (ANS) has been increasing with the deeper understanding of the underlying mechanisms [46, 47]. Stimulus- or condition-related HRV changes occur within the range of physiological variability and are undetectable without appropriate data processing in the time and frequency domains or by geometrical or nonlinear methods [48, 49]. Abnormal HRV measures of parasympathetic and sympathetic modulation are reportedly associated with increasing white matter lesion scores and mild cognitive impairment in traumatic brain injury (TBI) [50]. Functional damage and outcome correlate with HRV in TBI children, and HRV proved to be an independent predictor of outcome after rehabilitation from stroke in men, but not in women, while both HRV and the parasympathetic tone were significantly lower in adults with poor recovery than in those with good recovery. Brainstem damage and clinical worsening in TBI patients are associated with abnormal HRV measures, and sympathetic hyperactivity and overresponsiveness to afferent stimuli have been observed in a HRV study on TBI patients with paroxysmal sympathetic hyperactivity [51]. Recovery of consciousness in TBI patients is associated with reduced parasympathetic and increased sympathetic activities, which are described by HRV measures. The increase of HRV total power towards normal values within 3 mo from TBI was correlated with recovery of autonomic function in a prospective study. Comparable and replicable patterns of change in the significant HRV measures were observed in both healthy controls and TBI patients listening to classical music of different authorship aimed at evoking distinct emotional responses [52, 53]; comparable HRV responses were observed in VS/UWS. The HRV descriptor of sympathetic activity* nuLF* increased in the VS/UWS subjects while interacting with relatives (the jargonized* mom effect*) in the absence of HRV changes in control conditions [54]. The HRV is suitable in documenting residual emotional responsiveness and is monitored under proper conditions in all DOC subjects in the institute.

## 7. Responsiveness and the Central Autonomic Network

The autonomic nervous system governs a complex, highly differentiated network of distributed organs and biological sensors; it adjusts to or compensates for internal and external needs in the processes that collectively sustain internal environment constancy, adaptation, and homeostasis [55–57]. Neuroimaging studies have documented direct/indirect functional interaction between autonomic control and the brain activity in structures that are also involved in higher brain functions, possibly also including consciousness [45, 58]. To describe this interaction, a model network (the Central Autonomic Network, CAN) has been proposed according to which the anterior cingulate cortex and its projections to the prefrontal cortex, amygdala, hypothalamus, and brainstem are involved in the modulation of autonomic output in response to pain and emotional or behavioral stimulus conditions [59, 60]. Responsiveness appears to depend also on changes in the functional brain state that may occur spontaneously or may be induced by a variety of possible neuronal or nonneuronal factors, but major contributions by the ANS appear unquestionable. In recent studies, responsiveness proved to be correlated in VS/UWS with the sympathetic/parasympathetic functional balance as measured by HRV. The value intervals of HRV descriptors predicting with accuracy the highest incidence of response or no response at all have been experimentally defined [39] (Figure 3). Effects of factors modulating in parallel both the brain responsiveness and the autonomic balance or residual circadian/ultradian cycles asynchronous among subjects are possible [39] and these effects should be taken into proper account when assessing responsiveness in DOC.

## 8. Pain

Difficult to determine in noncommunicative subjects, pain is severe interference in the care and rehabilitation process of DOC subjects and needs continuous monitoring. The Nociception Coma Scale (NCS) is the only scale specifically assessing nociception in the severely brain-injured patients [61, 62] among the several scales in use or proposed for noncommunicative patients [63, 64]. It has been validated in a collaborative study by defining experimentally the interrater agreement, test-retest reliability, and diagnostic sensitivity [65]. In use in the institute, it allows a better discrimination between VS/UWS and MCS compared to the Newborn and Infant Pain Scale (NIPS) and the Pain Assessment in Advanced Dementia Scale (PAINAD).

## 9. Outcome

**Figure 4:**
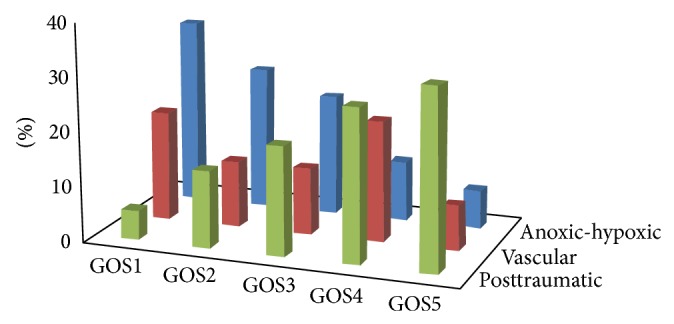
Overall outcome of patients in VS/UWS at admission in the years 1998–2013. The GOS classes are as follows: GOS1 = death; GOS2 = VS/UWS exceeding one year in duration; GOS3 = recovery, with severe disabilities; GOS4 = recovery, with mild disabilities; and GOS5 = full recovery or recovery with minimal disabilities not interfering with the everyday life [23].

Evolution from VS/UWS into MCS and outcome are evaluated by two established major descriptors, namely, the Glasgow Outcome Scale [66] and the observation of a visual pursuit response. The pursuit response is mediated in healthy subjects by activation of structures that are metabolically impaired in DOC subjects unable to sustain a visual pursuit [67], the reappearance of which is thus regarded as indicative of improvement and recuperation of the corticocortical and brainstem-cortex connectivity interfered with in VS/UWS and MCS [68, 69]. Posttraumatic patients have better outcome than vascular subjects and the outcome of anoxic-hypoxic subjects is the worst (Figure 4). A positive visual pursuit response was observed during follow-up in 81.8% of TBI and 64.7% and 35.7% of patients with massive vascular or anoxic-hypoxic brain damage, respectively. It was first observed within 50 days after brain injury in about 60% of TBI or vascular subjects and in 21% of anoxic-hypoxic patients, with maximum incidence at the end of follow-up (89% and 88% in TBI and vascular subjects, resp.; 67% in anoxic-hypoxic patients). Subjects whose pursuit response reappeared at any time during follow-up had better outcome (i.e., higher ratings at the GOS, usually in classes 4 and 5, at the end of follow-up) than those without it. The time of reappearance after brain injury inversely correlated with outcome; the correlation was higher among TBI than in vascular subjects. However, most vascular subjects with a pursuit response reappearing earlier than 100 days after brain injury reached GOS rating 2 at the end of follow-up (versus GOS 1 of those without response) [70]. It should be noted in this respect that subjects recovering while still in the process of being transferred from local hospitals to the institute and not in VS/UWS at admission had better GOS ratings at discharge than those in VS/UWS irrespective of etiology.

## 10. Continuous Monitoring by Ambient Intelligence

Continuous observation of VS/UWS and MCS subjects is mandatory to readily identify significant functional changes or markers of responsiveness that would help update diagnosis and prediction of outcome or adapt the rehabilitation program to individual needs. Continuous monitoring allows for the collection of large datasets but requires committed logistics, labor, time, and funding usually available only in large establishments. Alternative approaches based on advanced technology are available today. Among these,* ambient intelligence* (*AmI*) is a concept evolution of the Smart Environment Systems and takes advantage on other expert or artificial intelligence systems, that is, unobtrusive hardware, mobile/fixed communications infrastructure, dynamic and massively distributed device networks, and natural feeling human interfaces, dependability and security [71–73]. The Institute S. Anna-RAN has implemented an* AmI* platform with heterogeneous data acquisition systems (such as traditional or innovative sensor networks) and software modules [28, 74]. The platform provides tools for real-time automatic acquisition and processing of large datasets through pervasive but noninvasive hw/sw infrastructures. These allow two-way human/environment interactions at varying levels of functional complexity and the handling of incoming information about the specific characteristics of human presence and needs to which respond intelligently. The facility combines sensors for the ambient (temperature and humidity, CO_2_, light/dark cycles, and noise) and for each subject's relevant parameters (body temperature, heart rate and systolic/diastolic blood pressure, breathing, pO_2_, pCO_2_, spontaneous movements, voice, eye movements and eye blinking, and HRV). Spontaneous or stimulus-/event-related brain signals can be recorded as well. The environment and subjects' data are preanalyzed in biometric nodes. Raw and pretreated data are transferred wirelessly to a gateway for storage and further processing [74].

**Figure 5:**
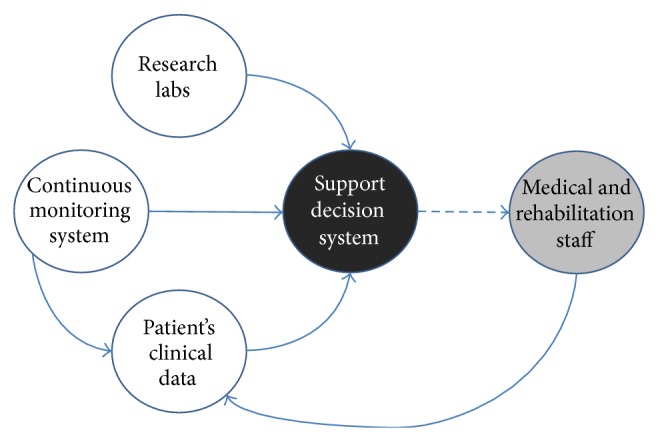
General scheme of the ambient intelligence system now operative at the Institute S. Anna-RAN.

*AmI* sets an innovative paradigm in information technology to improve (local or remote) individual care, with full integration of artificial intelligence technologies, data acquisition systems, and interfaces to potentiate the system intelligence through intensive and iterative processes aimed at identifying relevant correlations, trends, or patterns between the environment and the subject's parameters, including descriptors of brain function. In this respect, the* AmI* facility at the Institute S. Anna-RAN (Figure 5) complies with the standards of the international Integrating the Healthcare Enterprise (IHE) board and the eHealth HL7 format (http://www.ihe.net/; http://www.hl7.org/) as a hedge technological approach in the eHealth functional integration of biomedical and traditional domotics/informatics in hospital and home care.

## 11. Large Databases Analyses and Diagnostic Support

The datasets produced by* AmI* or otherwise are interfaced for compatibility and interplay with advanced tools for knowledge management and knowledge discovery purported to infer new knowledge from acquired and processed data [75]. Data mining techniques have also been implemented and are in use, at the interface of database technology, modeling techniques, statistical analysis, pattern recognition, and machine learning [76]. The system makes use of advanced tools for data management and automatic/semiautomatic analyses of large databases in order to identify significant trends and associations potentially informative because they are novel, implicit to the data, and of support in prediction and decision making. Other available algorithms are the regression analysis (to identify relationships among variables), the Neural Network (a sophisticated pattern detection algorithm using machine learning techniques to generate predictions), the Clustering/Segmentation processes (to create groups for applications), the Association Rules techniques (to detect related items in a dataset), and the Sequence Association tools (to detect causality and association between time-ordered events) [28, 74, 76].

## 12. Comment

The increased survival and better chances of favorable outcome have substantially modified the scenario of DOC [3, 77], the characteristics of which now appear compatible with a multifaceted condition—or a variety of conditions—that may develop independently from the etiology and are eventually masked by coma following brain injury [3, 77]. At the Institute S. Anna-RAN, substantial percentages of subjects in VS/UWS recovered consciousness and attained recuperation to levels compatible with autonomy or quasinormal or normal life. Only 12.6% of subjects with traumatic or vascular brain injury could be correctly diagnosed as still being in VS/UWS eight months after brain injury; late evolution into a MCS was observed in 3% of cases after up to two years from injury. Outcome proved more favorable in the subjects evolving earlier into a MCS [70]. Early care and neurorehabilitation are obviously crucial in this respect, particularly if a therapeutic continuum congruent to the functional brain organization at each phase during evolution from coma to VS/UWS to MCS to recovered consciousness is in operation. Professional commitment, logistics, and costs must be sustainable and hospitalization must be limited by time. The overall architecture of the Institute S. Anna-RAN has been originally designed [78] and upgraded in the following years in order to manage a sequence of activities to investigate residual brain function(s) in each subject with DOC and make use of any newly acquired knowledge to improve diagnosis and treatment in a circular, self-supporting process producing scientific, translational, and applicative research. In association with the turnover among units and home care under remote medical control of the eligible subjects, the approach has limited hospitalization to six months in almost the totality of cases.

Neuroimaging documents the capability of the severely damaged brain to express surviving modular functions despite impaired corticocortical and corticosubcortical connectivity [79, 80]. This evidence has been understood as indicative of retained (covert) cognition or consciousness as opposed to alternative interpretations that markers of neural activity are not necessarily surrogates for consciousness [31, 32]. The controversy challenges our definitions of phenomenal consciousness; the implications can entangle the current diagnostic criteria for VS/UWS as well as the medical care or legal or popular perception of bioethical issues, allocation of human resources and logistics, healthcare policies, and so forth. The pathophysiology of responsiveness and its clinical relevance and role in the classification and early prognosis of VS/UWS and MCS remain a practical problem while qualifying as a major scientific issue. In particular, the responsiveness variability (either spontaneous or in relation to the physiological or residual circadian/ultradian rhythms) supports the current diagnostic/prognostic criteria and helps characterize with greater accuracy the residual brain function(s). The identification in real time of the time windows during the day with better responsiveness helps define the optimal timing for neurorehabilitation [39]. To this end, responsiveness needs to be systematically tested by noninvasive technology compatible with continuous, sustainable monitoring.

Today, transferring promising advanced technology to a stage of regular operation in the neurorehabilitation of DOC subjects is a main goal in neurorehabilitation. To this end, domotic and robomechatronic devices and advanced informatics systems are being developed and validated in the framework of dedicated collaborative research projects. Contrary to expectation, the costs in time and labor are estimated to remain higher than in traditional therapy today and in the near future [81]. Crucial implications (such as organization of personnel, privacy concerns, or financial issues) are decisive in this context [81]; for this reason, pervasive but noninvasive systems (such as* AmI*) are privileged in the process. A major limit comes from this advanced technology being commonly conceived for application in neurorehabilitation as a substitute for traditional treatment, while knowledge on the pathophysiological processes involved in spontaneous recovery and rehabilitation unavailable in the past remains largely incomplete today [82]. The extensive application of advanced technology in research and clinical routine needs to result in novel treatments devised to overcome the traditional patient-therapist dichotomy and to transform neurorehabilitation into a self-training experience under proper guidance [82]. Flexible, highly specialized options including sensory/cognitive/emotional interaction able to adjust to individual necessities and changes during treatment should be devised. Flexibility in the general architecture, organization, and planning of the hosting institution (and its extensions for monitored home care) is mandatory in order to meet the—often unpredictable—changes in the natural history of the disorder, patients' clinical needs, and healthcare policy.

## Figures and Tables

**Table 1:** Demographic table years: 1998–2013.

Subjects	*n* (%)	Sex M/F (%)	Age, years mean (SD)	Time in intensive care unit before admission, days mean (SD)	Time in the semi-intensive care unit, days mean (SD)	Time in brain injury care unit, days mean (SD)
All patients	1068	764/304	46 (20)	43 (33)	55 (46)	77 (63)
Others	21 (2)	7/14	56 (15)	52 (43)	43 (42)	45 (44)
Anoxic patients	68 (6)	40/28	52 (19)	49 (46)	79 (51)	98 (93)
TBI patients	523 (49)	432/91	34 (18)	40 (28)	53 (46)	68 (62)
Vascular patients	456 (43)	286/170	58 (16)	45 (34)	55 (46)	84 (84)
